# Psychosocial Burden of Multiple IgE-Mediated Food Allergies in Pediatric Patients and Caregivers in the FORWARD Study

**DOI:** 10.3390/nu18111745

**Published:** 2026-05-29

**Authors:** Mariesa Cay, Caglar Onal, Linda Herbert, Melissa Engel, Hemant Sharma, Mahdavinia Mahboobeh, Amal Assa’ad, James Moy, Lucy Bilaver, Ruchi Gupta, Christopher Warren

**Affiliations:** 1 Center for Food Allergy and Asthma Research, Northwestern University Feinberg School of Medicine, 750 North Lake Shore Drive, 6th Floor, Chicago, IL 60611, USA; 2 Children’s National Hospital Medical Center, Washington, DC 20010, USA; 3 University of Texas Health Science Center, Houston, TX 77030, USA; 4 Cincinnati Children’s Hospital Medical Center, Cincinnati, OH 45229, USA; 5 Rush University School of Medicine, Chicago, IL 60612, USA; 6 Ann and Robert H Lurie Children’s Hospital of Chicago, Chicago, IL 60611, USA

**Keywords:** food allergy-related quality of life, psychosocial burden, anxiety, food allergy management, multiple food allergies, food allergy burden

## Abstract

**Background/Objectives**: Of the approximately 8% of children in the United States (US) with food allergy (FA), roughly 40% report allergy to multiple foods. The ubiquity and intensity of FA management can impose psychosocial burdens on both pediatric patients and caregivers, which may be exacerbated among those allergic to multiple foods. However, little work has comprehensively estimated the burden of multi-FA in heterogeneous pediatric populations with clinically confirmed FA. **Methods**: Children with allergist-diagnosed, IgE-mediated FA were enrolled in the FORWARD multisite prospective cohort study. Psychosocial burden was assessed annually using the FA Quality of Life Questionnaire-Parent Form 10 (FAQL-PF10) and FA Independent Measure-Parent Form (FAIM-PF); psychosocial burden for caregivers was measured with the FA Quality of Life-Parental Burden (FAQL-PB) Questionnaire. Multilevel regression models estimated independent effects of the number of current FAs after adjusting for child age, gender, race, ethnicity, household income, caregiver education, atopic comorbidities, and recruitment site. **Results**: FA-related psychosocial burden increased linearly for patients and caregivers with each additional FA even after controlling for demographic factors such as age, gender, race, ethnicity, income, education, and other atopic conditions, as well as repeated observations within participants: FAQL-PF10 B = 0.082 (*N* = 2206; *p* = 0.004), FAIM-PF B = 0.152 (*N* = 2206; *p* < 0.001), and FAQL-PB B = 0.166 (*N* = 1081; *p* < 0.001). **Conclusions**: The psychosocial burden of pediatric FA increases monotonically with each additional current FA, highlighting opportunities for tailored psychosocial, behavioral, and pharmacologic interventions to improve FA-related outcomes among those most impacted.

## 1. Introduction

IgE-mediated food allergy (FA) is a growing public health concern that affects approximately 8% of children [1]. FA can lead to severe and potentially fatal reactions, and thus requires constant and meticulous avoidance of allergens, a burden that can impact quality of life in both patients and their caregivers [2]. Current management mainstays either require strict allergen avoidance or therapeutic approaches that can impose a high treatment burden. As a result, daily activities such as eating in the school cafeteria, going out to a restaurant, or attending a birthday party require advance planning that can incur social and financial burdens that cause many families to avoid these activities altogether [3]. Even with careful planning intended to ensure allergen avoidance, food allergens may appear unexpectedly: from M&Ms used for demonstrations in math class to an impromptu ingredient swap in a grandparent’s cookie recipe. To mitigate risk, children with FA and/or their caregivers must take on the responsibility of always carrying epinephrine and ensuring that they and those around them are educated in its use, further adding to the burden of living with FA.

US population-based survey data estimate that among children with an IgE-mediated FA, 40% have multiple FAs [4]. Prior studies have observed decreased quality of life for both patients with multi-FA [5,6,7] and their caregivers [8,9,10,11]. For example, when using the Food Allergy Quality of Life-Parental Burden (FAQL-PB) questionnaire to compare caregivers of children with single FA vs. multi-FA, those with multi-FA displayed a greater burden overall, as well as in each of the 17 assessed subdomains [10,11]. Nonetheless, the samples analyzed to date have been small, and homogenous, and most data are over ten years old, leading to concerns about their generalizability to today’s FA management landscape. For example, in the past decade alone, there have been increased rates of FA diagnosis, new management approaches, FDA-approved immunotherapies, NIAID-endorsed FA prevention guidelines, and broader societal awareness with more allergen labeling and allergy-safe food alternatives available in many grocery stores and restaurants.

Furthermore, given that at least 40% of FA patients are allergic to multiple foods, it is also important to quantify the heterogeneity in burden among this multi-food-allergic subpopulation. Prior works suggest that children with more FAs appear at higher risk of worse outcomes including FA-related emergency department visits [4], and food-induced anaphylaxis [12]. However, the psychosocial burden imposed by multi-FA is less clear as most studies to date have relied on a binary classification of single FA vs. multiple FA. This is important because clinical observation suggests that, for example, a patient with allergy to peanut, tree nut, milk, egg, soy, and wheat would generally carry a much greater burden than a patient with an allergy to peanuts and cashews alone. However, to date no studies have examined this phenomenon in a sufficiently large sample of US children with clinically confirmed FA to draw meaningful inferences about the psychosocial burden of multi-FA—particularly its manifestations across key racial and ethnic minority subpopulations (e.g., Black or Hispanic children and their caregivers) who have been historically underrepresented in studies of the lived experiences of those affected by food allergy [13].

The present study aims to leverage data collected from the large, racially, ethnically, socioeconomically and geographically diverse FORWARD cohort to estimate the FA-related psychosocial burden associated with increasing numbers of current FAs among US children with multi-FA and their caregivers. Given what is known about psychosocial burden in single vs. multi-FA [6,8,9,10,11,14] as well as adverse health outcomes associated with having multiple FA [12], it is hypothesized that FA-related psychosocial burden will increase linearly with each additional FA for both pediatric patients and their caregivers.

## 2. Materials and Methods

Data from the present study were collected as part of the FORWARD study R01-AI130348, a longitudinal cohort of children recruited when they were 12 years old or younger with a physician-diagnosed FA from the greater Chicago (Ann & Robert H. Lurie Children’s Hospital, Rush University Medical Center), Cincinnati (Cincinnati Children’s Hospital), and Washington, D.C. (Children’s National Hospital) metropolitan regions between 2017 and 2025. To be recruited into the study, the child (12 years old or younger at the time of the intake visit) must have at least one physician-diagnosed IgE-mediated food allergy diagnosed by their allergist based on a documented allergic reaction to the eliciting food and evidence for sensitization. An allergic reaction was defined as a reaction occurring within two hours of ingesting the suspected food and may include cutaneous, gastrointestinal, respiratory, cardiovascular, or multi-systemic symptoms. Evidence of IgE sensitization to each documented food allergen was confirmed through either a positive skin prick test (defined as a wheal ≥ 3 mm above the negative control and/or via food allergen-specific IgE > 0.35 kU/L The methodology of FORWARD has previously been described [15] and all human subjects research activities were IRB-approved. Informed consent was obtained from all subjects involved in the study.

### 2.1. Study Outcomes

Patient-level FA-related psychosocial burden was assessed by the following three caregiver-proxy reported measures, each of which has been validated for use in pediatric samples. The Food Allergy Quality of Life Questionnaire-Parent Form 10 (FAQL-PF10) [16] was administered every 12 months starting at 3 months after enrollment. The FAQL-PF10 is a 10-item questionnaire to assess feeling different from other children, reluctance to try new foods, emotional distress, food limitations, restaurant and travel/vacation limitations, worry around new people, social frustration, cautiousness, desire for FA to go away, and concern with likelihood of future reactions. Each item is assessed on a Likert scale from 0 to 6 with lower scores representing better quality of life. The composite measure represents the mean of all 10 items.

The Food Allergy Independent Measure-Parent Form (FAIM-PF) [17] was administered every 12 months starting at 3 months after enrollment. The FAIM-PF is a seven-item questionnaire consisting of four items assessing “Expectation of Outcome”—specifically the perceived chance of accidental allergen exposure, chance of reaction, chance of death, and chance that child or those around them will have knowledge on how to appropriately manage an acute reaction in the event of allergen exposure. The FAIM also includes three items assessing FA-related dietary limitation as well as FA-related social limitations for the child and the broader family unit. Each item is measured on a Likert-type scale from 0 to 6 with lower scores representing lower burden. The composite score represents the mean of all seven items.

A domain general (i.e., global) caregiver-reported health measure—the PROMIS Pediatric Global Health Parent Proxy (PGH-7) [18]—was administered at 56 months after enrollment. This PROMIS measure is a seven-item caregiver report to assess a child’s general health, quality of life, physical health, mental health, sadness, fun with friends, and perception that caregivers listen to their ideas. Each item is measured on a Likert scale from 1 to 5 where lower scores represent better health. The composite score represents the mean of all seven items.

Finally, a self-report measure of FA-related caregiver burden, the Food Allergy Quality of Life-Parental Burden (FAQL-PB) [9], was administered at 9 months and 21 months after enrollment. The FAQL-PB is a 17-item questionnaire with one item assessing limitations for each domain of travel/vacation, dining, social activities, meal preparation, leaving the house, anxiety, overcoming allergies, trusting others, appreciation for severity, sadness, school/camp, health concerns, ability to help, normalcy, nutrition, eating with others, and fear of reaction. Each item is measured on a Likert scale of 0 to 6 where higher scores represent increased caregiver burden. The composite score represents the mean of all 17 items.

### 2.2. Covariates

Demographic information was obtained from the FORWARD intake survey. Child race was categorized as White, Asian, Black, or Other based on caregiver-proxy response to the intake survey item, “With which race does your child with food allergy primarily identify?” Hispanic ethnicity was assessed by a separate item. Other demographic variables included male or female child gender, current child age in years, parent education, annual household income, and recruitment site.

Comorbid atopic conditions, including physician-diagnosed asthma, eczema, allergic rhinitis, and oral allergy syndrome (OAS), were also included as covariates due to their potential contribution to psychosocial burden.

The total number of current “top 9” food allergens (i.e., peanut, tree nut, milk, egg, wheat, shellfish, fin fish, soy, and sesame), as well as a tenth “other” category was calculated per child based on a combination of allergist testing, electronic health record data extraction, and survey data.

### 2.3. Statistical Analyses

Descriptive statistics summarized demographics as well as individual survey items. Multilevel linear regression models were fit in R version 4.5 using lme4 to determine the independent effects of a one unit increase in total number of current FA on each measured outcome, adjusting for the above covariates, which were specified a priori and modeled comparably in prior work [19]. To account for potential clustering due to multiple children from the same household (13% of the analytic sample), random intercepts for household nested within institution were included. Repeated measures within the same participant were accounted for by including patient-level random intercepts and slopes. All statistical tests were two-sided with an alpha level of 0.05.

## 3. Results

### 3.1. Demographics and Clinical Atopic Disease Characteristics

Given that questionnaires assessing psychosocial outcomes were administered at different points throughout the course of this longitudinal cohort study, demographic information for each measure varies slightly. Values reported here and in Table 1 reflect all available data for the FAQL-PF10 and FAIM questionnaires, which were administered together starting at 3 months post-enrollment—the earliest assessment of FA-related psychosocial outcomes in the cohort and therefore the most available responses. Demographic information for other measures can be found in Table A1 and Table A2.

In summary, our analytic sample demographically comprised roughly 62% males and had a mean age of approximately 6 years at the time of their first completed psychosocial survey. Older children were significantly more likely to be allergic to a greater number of foods. The racial distribution of the sample was approximately 50% White, 37% Black, 5% Asian, and 8% other, while about 20% of the sample reported Hispanic ethnicity. Children of White race and children of Hispanic ethnicity were less likely to have a greater number of food allergies, whereas children of Black race were more likely to have a greater number of food allergies. Additionally, 45% of the sample reported an annual pre-tax household income of <$100,000 with approximately 1/3 of households earning at least $200,000/year. Most caregivers (87%) reported at least a semester of college education.

With respect to clinical food allergy characteristics, peanut was the most common food allergy, followed by any tree nut allergy, hen’s egg, cow’s milk and shellfish. Over 80% of the sample had a history of eczema, while 47% were diagnosed with allergic rhinitis, 42% had asthma, and 39% had been diagnosed with oral allergy syndrome. Each atopic comorbidity was more common among children with an increasing number of food allergies.

### 3.2. Child Psychosocial Burden

The dataset contained 2206 observations from 967 participants for the FAQL-PF10 and the FAIM. As seen in Table 2, there is a significant difference in mean score for single vs. multi-FA for all 10 of the items on the FAQL-PF10 as well as the composite score. Furthermore, there was a significant difference in mean score for single vs. multi-FA for all seven items of the FAIM and the composite score, with relatively higher scores observed for patients with multi-FA.

As seen in Table 3, when examining single vs. multi-FA as a categorical variable in the regression models, there is a significant increase in burden for multi-FA in 5 of the 10 domains and the composite score (B_cat_ = 0.233, *p* = 0.008). The FAIM regression models for single vs. multi-FA are significant in all seven domains and the composite score (B_cat_ = 0.400, *p* < 0.001), again indicating a positive association between more FAs and higher FAIM scores.

**Figure 1:**
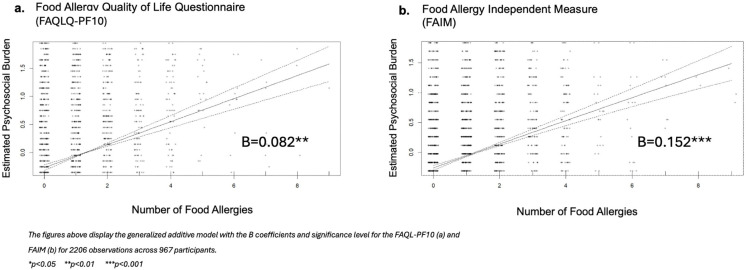
Child burden composite score regression models. The figures above display two generalized additive models with B coefficients and significance levels for the FAQLQ-PF10 and FAIM. The solid lines reflect the fitted GAM smooths, whereas the dashed lines reflect an approximate 95% pointwise confidence band around those fitted GAM smooths, representing the estimated mean relationship between number of food allergies and each measure of FA-related psychosocial burden. * *p* < 0.05; ** *p* < 0.01; *** *p* < 0.001.

Also shown in Table 3, when examining the total number of current FA as a continuous variable, the regression models are significant for 7 of the 10 items on the FAQLQ-PF10 and the composite score (B_cont_ = 0.082, *p* = 0.004; see Figure 1a)—indicating a positive association between FAQLQ-PF10 scores and multi-FA. Likewise, for the FAIM, the continuous variable regression models are significant and positively associated for all seven items and the composite score (B_cont_ = 0.152, *p* < 0.001; see Figure 1b). Note that the beta coefficients reported in the text are from linear mixed-effects models. Figure 1a,b illustrates smoothed, non-linear relationships estimated separately using generalized additive models (GAMs) for visualization purposes. The GAM plots support an approximately linear relationship, validating the linearity assumption of the mixed-effects models.

### 3.3. Global/Domain-General Health

**Figure A1:**
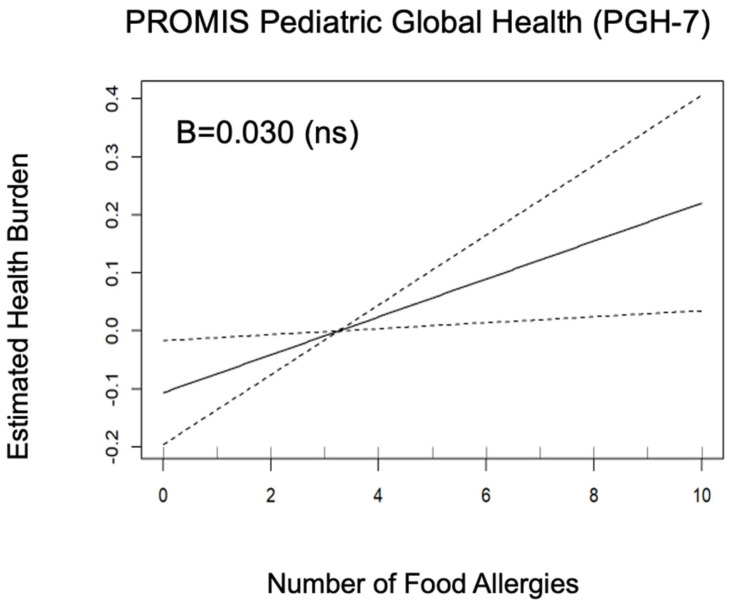
Global Health Composite Score Regression Model. *The figures above display the generalized additive model with the B coefficient and significance level for the PROMIS Pediatric Global Health (PGH-7) for 333 observations. ns = not significant.*

The dataset contained 333 observations for the PGH-7. Composite scores and significant domains are described here; detailed results can be found in Table A3 and Table A4 and Figure A1. When examining single vs. multiple FA, there is a significant difference in mean score for mental health (*p* = 0.044)—with greater mental health burden positively associated with multi-FA. However, no significant differences were observed for any of the other domains or composite score. For the regression model with multiple FA as a categorical variable, there is no significant difference between single and multi-FA (B_cat_ = 0.108, *p* = 0.272) in the composite global health score. When estimating the linear effect of additional FA, parameterized as a continuous variable, there is no significant increase in global health burden with each additional allergen (B_cont_ = 0.030, *p* = 0.133). However, parents of children with an increased number of allergies were more likely to report that the child felt their parents do not listen to their ideas compared to parents of children with fewer allergies (B_cont_ = 0.101, *p* = 0.032).

### 3.4. Parent Psychosocial Burden

**Figure 2:**
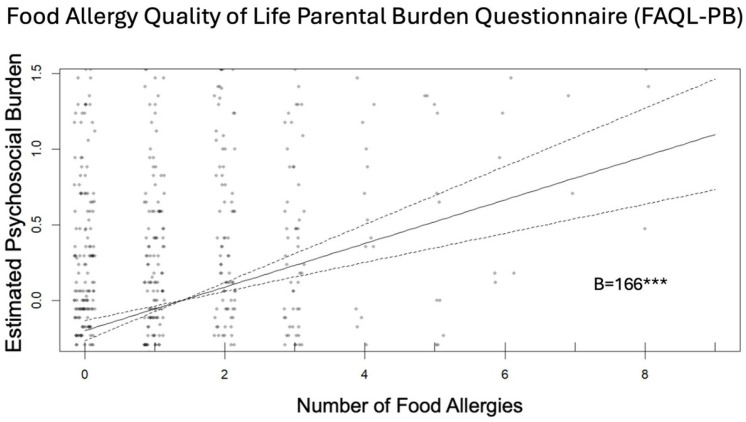
Parent burden composite score regression model. The figure above displays the generalized additive model with the B coefficient and significance level for the parental burden questionnaire. The solid line reflects the fitted GAM smooth, whereas the dashed lines reflect an approximate 95% pointwise confidence band around the fitted GAM smooth, representing the estimated mean relationship between number of food allergies and parental FA-related psychosocial burden. * *p* < 0.05; ** *p* < 0.01; *** *p* < 0.001.

For the FAQL-PB, 1081 observations were obtained from 729 caregivers. As seen in Table 4, there is a significant difference in the mean score for single vs. multi-FA for all 17 items of the FAQL-PB as well as the composite FAQL-PB score, with greater FAQL-PB scores positively associated with multi-FA. As shown in Table 5, when examining single vs. multi-FA as a categorical variable in the regression models, there is a significant increase in burden among caregivers of patients with multi-FA on all 17 domains as well as the composite score (B_cat_ = 0.567, *p* < 0.001). Furthermore, as visualized in Figure 2, when examining parental burden with total number of allergies as a continuous variable, there is also a significant increase in parental burden among caregivers of children with multi-FA in all 17 domains and the composite score (B_cont_ = 0.166, *p* < 0.001).

## 4. Discussion

There exists a high population-level burden of FA in the United States and globally [20] and the growing array of therapeutic options for FA patients and their families such as food allergen immunotherapies and psychosocial supports. Thus, it is crucial to identify patients who can potentially experience the greatest benefit to maximize available public health resources. Data from the present study suggest that from a parent perspective, children with more current FAs experience higher FA-related psychosocial burden and lower FA-related quality of life, as do their caregivers. These findings were consistent across each of the three validated FA-specific psychosocial measures administered as part of this study—the FAQL-PF10, FAIM-PF, and FAQL-PB—and were robust to adjustment for an extensive set of relevant covariates, which include age, gender, race, ethnicity, household income, parent education, comorbid atopic conditions, and recruitment site. These data suggest that a difference of 2 FAs (e.g., going from 2 to 4 FAs) may approximate a minimally clinically important difference based on prior work by the authors of the FAQLQ-PF [21] and FAIM as well as others [22].

To our knowledge, this is the first study that has aimed to estimate the psychosocial burden of multi-FA in such a large, racially, ethnically, and socioeconomically diverse sample of children with allergist-confirmed FA and their caregivers. Given increasing acknowledgement of the heterogeneity of FA phenotypes and the potential importance of more tailored approaches to FA treatment and management, this study is also notable in its estimation of the psychosocial burden associated with increasing numbers of FAs, finding the effects of additional FA to be well characterized by a linear model. In studies to date, multi-FA is most often modeled as a binary single vs. multiple FA variable. However, the present data indicate an additive, monotonic relationship between FA-specific psychosocial burden and the number of current FA—at least within this sample of patients with allergist-diagnosed FA seeking care at allergy clinics affiliated with four large urban/suburban academic medical centers. For example, the domains of emotional distress from allergic reactions, feeling frustrated by restrictions, and wishing FA would go away were not significant when examining single vs. multi-FA as a categorical variable, but all three were significant when examining total allergies as a continuous variable. This highlights that there is substantial explanatory value in considering the number of current FAs as a continuous variable when modeling psychosocial outcomes for children and families living with FA.

Importantly, to help rule out the alternative explanation for our findings that children with more current FA experience poorer health outcomes more generally (i.e., not just in the domain of FA), we also modeled parent proxy-reported pediatric QoL as assessed by the extensively validated PGH-7. No significant associations were found between single vs. multi-FA and global health; but there was a reliably observed small increase in adverse global quality of life impact in each PGH-7 subdomain. This suggests that while FA leads to FA-specific burden, at a certain point, FA-specific burden may contribute negatively to overall wellbeing, and more general impairments in reported physical health and social interactions. However, further work employing both FA-specific and domain general QoL scales in larger samples of FA patients is needed to draw more firm conclusions. As seen in Table 1, the children in the present sample with more FAs also have higher rates of specific atopic comorbidities, the severity of which may also adversely contribute to patient and caregiver quality of life.

Limitations of the present study include its reliance upon an allergist-referred patient population seeking care at four major urban/suburban University-affiliated pediatric allergy care networks, whose FA-related psychosocial burden may be greater relative to the general food-allergic pediatric population in the US. It is also notable that compared to the US pediatric population, the present analysis includes a relatively low number of Hispanic/Latino patients, which made it difficult to assess how the psychosocial burden of multiple FA may differ in this population. However, the FORWARD study began recruiting Hispanic/Latino families in 2022, so more information on this population (who can complete assessments in English and Spanish) will be available as the cohort continues accruing data. Likewise, the present study only includes caregiver-report data, and prior research has shown that children tend to perceive a higher FA burden than what their parents report [23,24]. This may be especially the case in younger children who may not be able to fully communicate their feelings, and older adolescents [7] who may be more independent in managing their FA outside of direct parental oversight. Ongoing collection of complementary child-reported psychosocial outcomes, which was initiated in the FORWARD study for adolescent participants in 2022, will allow future research to more systematically incorporate children’s own perspectives on the psychosocial burden of living with multiple FA and compare child vs. parent-proxy report of FA-related psychosocial burden across development and other relevant patient demographic and clinical phenotypes. Finally, for parsimony and consistency with prior work, we decided to group all tree nuts, fin fish, and shellfish allergies under these superordinate categories. While our currently employed approach to modeling these allergen groups categorically attempts to avoid disproportionately inflating allergen counts of patients with multiple botanically related allergies (e.g., many seafoods that all share the same eliciting protein—tropomyosin; or commonly cross-reactive tree nuts like cashew/pistachio), it has two chief limitations. First, it may underestimate the true heterogeneity of FA burden among patients and therefore flatten a true dose–response relationship between FA burden and the number of specific tree nut/shellfish/finned fish allergies a patient has. Second, it may create a non-differential exposure misclassification whereby children with very different numbers of specific allergies are assigned the same value of “tree nut-allergic” or “shellfish-allergic”, thereby biasing associations toward the null if a dose–response relationship truly exists. Beyond these three categories, it is also important to acknowledge that different food allergies are likely to impose distinct burden—as the impact of FA on daily life can reasonably be assumed to vary based on the ubiquity of each allergen in the social and home environments as well as the heterogeneous population-level immunologic dynamics of each food antigen (e.g., typical reaction severity, threshold eliciting dose). For this reason, future work will attempt to examine specific tree nuts, fin fish, and shellfish as distinct allergies, with corresponding psychosocial and management impacts, since a patient with true IgE-mediated allergies to many individual tree nuts may indeed be more likely to have increased daily burdens associated with additional allergen avoidance relative to another patient with allergy to only a single tree nut.

## 5. Conclusions

In conclusion, this is the first study to show an increased psychosocial burden associated with each additional FA in both children and their caregivers using multiple validated measures in a sample of US patients with clinically confirmed FA. Future work should focus on the psychosocial burden of multiple FA in Hispanic/Latino populations and from child perspectives, which ongoing data collection in the FORWARD study will allow.

## Figures and Tables

**Table 1:** Demographic information for child burden measures.

	Total (N = 967)		1 FA (N = 217)		2–3 FA (N = 436)		4–6 FA (N = 256)		7+ FA (N = 58)		*p* Value
	N	%	N	%	N	%	N	%	N	%	
Age											
Mean Age (months) (S.D.)	69.9 (43.0)	-	56.7 (40.28)	-	70.1 (42.45)	-	75.2 (43.90)	-	90.7 (35.36)	-	<0.001 ***
Sex											0.437
Male	603	62.4	129	59.4	267	61.2	169	66	38	65.5	
Female	364	37.6	88	40.6	169	38.8	87	34	20	34.5	
Race											<0.001 ***
White	481	49.7	104	47.9	213	48.9	143	55.9	21	36.2	
Black	354	36.6	79	36.4	150	34.4	89	34.8	36	62.1	
Asian	45	4.7	10	4.6	27	6.2	8	3.1	0	0	
Other	78	8.1	19	8.8	43	9.9	15	5.9	1	1.7	
Ethnicity											<0.001 ***
Not Hispanic	743	76.8	153	70.5	328	75.2	212	82.8	50	86.2	
Hispanic	189	19.5	56	25.8	93	21.3	35	13.7	5	8.6	
Household Income											0.036 *
Less than $100,000	438	45.3	111	51.2	192	44	101	39.5	34	58.6	
$100,000–$199,000	215	22.2	39	18	95	21.8	70	27.3	11	19	
More than $199,000	314	32.5	67	30.9	149	34.2	85	33.2	13	22.4	
Parental Education											0.529
Some High School	34	3.5	9	4.1	12	2.8	10	3.9	3	5.2	
High School Grad	89	9.2	17	7.8	45	10.3	19	7.4	8	13.8	
Some College or Higher	844	87.3	191	88	379	86.9	227	88.7	47	81	
Allergens											<0.001 ***
Egg	511	52.8	50	23	198	45.4	205	80.1	58	100	
Milk	294	30.4	15	6.9	92	21.1	137	53.5	50	86.2	
Peanut	641	66.3	69	31.8	292	67	223	87.1	57	98.3	
Treenut	576	59.6	44	20.3	250	57.3	225	87.9	57	98.3	
Finfish	165	17.1	7	3.2	48	11	70	27.3	40	69	
Shellfish	186	19.2	14	6.5	54	12.4	77	30.1	41	70.7	
Wheat	109	11.3	1	0.5	14	3.2	51	19.9	43	74.1	
Soy	118	12.2	1	0.5	18	4.1	52	20.3	47	81	
Sesame	167	17.3	7	3.2	38	8.7	86	33.6	36	62.1	
Other	172	17.8	9	4.1	49	11.2	82	32	32	55.2	
Comorbidities											<0.001 ***
Asthma	414	42.8	67	30.9	184	42.2	117	45.7	46	79.3	
Eczema	804	83.1	154	71	366	83.9	232	90.6	52	89.7	
Allergic Rhinitis	451	46.6	60	27.6	190	43.6	154	60.2	47	81	
Oral Allergy Syndrome	381	39.4	49	22.6	156	35.8	133	52	43	74.1	

The table above displays the demographic information for the child burden measures (FAQL-PF10 and FAIM) for the total 967 participants and then also broken down into categories of children with 1 FA, 2–3 FA, 4–6 FA, and 7+ FA. * *p* < 0.05; ** *p* < 0.01; *** *p* < 0.001.

**Table 2:** Mean values of psychological burden in children with single FA vs. multiple FA.

	Single FA Mean (SD)	Multi FA Mean (SD)	Bivariate *p* Value
FAQLQ			
feels different from others	1.54 (1.58)	2.43 (1.72)	<0.001 ***
reluctant to try new foods	2.35 (1.87)	2.99 (1.92)	<0.001 ***
emotional distress from reaction symptoms	1.60 (1.85)	2.25 (1.86)	<0.001 ***
limitations on amount of safe foods	2.07 (1.71)	3.07 (1.81)	<0.001 ***
limitations on restaurants/vacations	1.63 (1.61)	2.89 (2.01)	<0.001 ***
worries in social situations	1.34 (1.72)	2.09 (1.86)	<0.001 ***
frustrated by restrictions	1.18 (1.56)	1.84 (1.77)	<0.001 ***
more cautious than other children	2.41 (2.08)	3.26 (2.01)	<0.001 ***
wishes FA was gone	2.59 (2.38)	3.36 (2.24)	<0.001 ***
concern about future reactions	1.77 (1.93)	2.46 (1.95)	<0.001 ***
composite	1.85 (1.38)	2.66 (1.43)	<0.001 ***
FAIM			
perceived chance of accidental ingestion	1.95 (1.41)	2.20 (1.39)	<0.001 ***
perceived chance of severe reaction	2.89 (1.78)	3.56 (1.69)	<0.001 ***
perceived chance of death	1.70 (1.62)	2.22 (1.61)	<0.001 ***
perceived chance of child not knowing what to do	1.80 (1.64)	2.15 (1.55)	<0.001 ***
amount of foods child is unable to eat	2.09 (1.50)	3.02 (1.38)	<0.001 ***
how much FA affects things child does with others	1.29 (1.36)	2.16 (1.73)	<0.001 ***
how much FA affects things family does together	1.14 (1.24)	2.02 (1.71)	<0.001 ***
composite	1.84 (1.06)	2.48 (1.09)	<0.001 ***

The table above displays the mean and *p*-values for single FA vs. multiple FA for the FAQL-PF10 and FAIM which are scores from 0 to 6 for 2206 observations across 967 participants. * *p* < 0.05; ** *p* < 0.01; *** *p* < 0.001.

**Table 3:** Psychological burden for children with multiple FA regression analysis.

	Categorical B Coefficient	95% CI Lower	95% CI Upper	Categorical *p* Value	Continuous B Coefficient	95% CI Lower	95% CI Upper	Continuous *p* Value
**FAQLQ**								
feels different from others	0.339	0.108	0.569	0.004 **	0.105	0.029	0.182	0.007 **
reluctant to try new foods	0.245	−0.02	0.511	0.070	0.084	−0.003	0.171	0.059
emotional distress from reaction symptoms	0.231	−0.02	0.482	0.071	0.104	0.022	0.186	0.0133 *
limitations on amount of safe foods	0.571	0.337	0.806	<0.001 ***	0.2	0.121	0.28	<0.001 ***
limitations on restaurants/vacations	0.573	0.339	0.808	<0.001 ***	0.225	0.15	0.3	<0.001 ***
worries in social situations	0.336	0.096	0.576	0.006 **	0.104	0.026	0.182	0.009 **
frustrated by restrictions	0.227	−0.008	0.463	0.059	0.069	−0.008	0.145	0.078
more cautious than other children	0.24	−0.021	0.502	0.071	0.119	0.038	0.201	0.0041 **
wishes FA was gone	0.094	−0.173	0.36	0.490	0.041	−0.049	0.132	0.371
concern about future reactions	0.307	0.061	0.554	0.015 *	0.085	0.002	0.169	0.0446 *
composite	0.233	0.062	0.403	0.008 **	0.082	0.026	0.137	0.004 **
**FAIM**								
perceived chance of accidental ingestion	0.333	0.151	0.516	<0.001 ***	0.113	0.049	0.176	<0.001 ***
perceived chance of severe reaction	0.451	0.231	0.672	<0.001 ***	0.137	0.069	0.206	<0.001 ***
perceived chance of death	0.228	0.006	0.449	0.044 *	0.098	0.026	0.17	0.007 **
perceived chance of child not knowing what to do	0.361	0.149	0.573	<0.001 ***	0.126	0.057	0.194	<0.001 ***
amount of foods child is unable to eat	0.573	0.391	0.755	<0.001 ***	0.251	0.193	0.309	<0.001 ***
how much FA affects things child does with others	0.567	0.347	0.787	<0.001 ***	0.213	0.138	0.288	<0.001 ***
how much FA affects things family does together	0.537	0.317	0.757	<0.001 ***	0.195	0.12	0.269	<0.001 ***
composite	0.4	0.261	0.54	<0.001 ***	0.152	0.106	0.197	<0.001 ***

The table above displays the B coefficient and *p*-values for categorical (i.e., single FA vs. multiple FA) and continuous (i.e., specific number of FA) for the FAQL-PF10 and FAIM for 2206 observations across 967 participants. * *p* < 0.05; ** *p* < 0.01; *** *p* < 0.001.

**Table 4:** Mean values of psychological burden in parents of children with single FA vs. multiple FA.

	Single FA Mean (SD)	Multi FA Mean (SD)	Bivariate *p* Value
Parental Burden Questionnaire			
limitations on family vacation	1.20 (1.52)	1.95 (1.86)	<0.001 ***
limitations on family dining at restaurants	1.85 (1.68)	2.81 (1.94)	<0.001 ***
limitations on family social activities	1.58 (1.56)	2.23 (1.74)	<0.001 ***
troubled with meal prep	0.85 (1.18)	1.33 (1.48)	<0.001 ***
troubled with special precautions	0.83 (1.24)	1.20 (1.43)	<0.001 ***
troubled with anxiety about child’s FA	0.84 (1.27)	1.25 (1.47)	<0.001 ***
troubled that child may not overcome FA	1.17 (1.54)	1.60 (1.71)	<0.001 ***
troubled with leaving child in care of others	1.16 (1.54)	1.65 (1.81)	<0.001 ***
troubled by other’s lack of knowledge of FA	1.05 (1.52)	1.35 (1.68)	0.004 **
troubled by sadness due to child burden	0.92 (1.38)	1.45 (1.70)	<0.001 ***
troubled by child’s attendance at school and extracurricular activities	0.95 (1.50)	1.37 (1.68)	<0.001 ***
troubled with concerns for child’s health	0.93 (1.39)	1.40 (1.65)	<0.001 ***
troubled with worry of inability to help in a reaction	1.06 (1.47)	1.29 (1.55)	0.021 *
troubled with worry child will not have normal life	0.78 (1.21)	1.17 (1.54)	<0.001 ***
troubled by concerns about child’s nutrition	0.63 (1.17)	1.22 (1.68)	<0.001 ***
troubled with child being near others while eating	0.99 (1.44)	1.35 (1.66)	<0.001 ***
troubled with fright about child having a reaction	1.13 (1.46)	1.46 (1.65)	0.001 **
composite	1.05 (1.14)	1.54 (1.32)	<0.001 ***

The table above displays the mean and *p*-values for single FA vs. multiple FA for the parental burden questionnaire, which is scored from 0 to 6 with 1081 observations across 729 participants. * *p* < 0.05; ** *p* < 0.01; *** *p* < 0.001.

**Table 5:** Psychological burden for parents of children with multiple FA regression analysis.

	Categorical B Coefficient	95% CI Lower	95% CI Upper	Categorical *p* Value	Continuous B Coefficient	95% CI Lower	95% CI Upper	Continuous *p* Value
Parental Burden Questionnaire								
limitations on family vacation	0.756	0.466	1.047	<0.001 ***	0.223	0.135	0.312	<0.001 ***
limitations on family dining at restaurants	0.722	0.407	1.037	<0.001 ***	0.183	0.087	0.28	<0.001 ***
limitations on family social activities	0.668	0.379	0.956	<0.001 ***	0.178	0.089	0.267	<0.001 ***
troubled with meal prep	0.503	0.259	0.748	<0.001 ***	0.17	0.095	0.245	<0.001 ***
troubled with special precautions	0.54	0.298	0.781	<0.001 ***	0.201	0.126	0.275	<0.001 ***
troubled with anxiety about child’s FA	0.596	0.347	0.844	<0.001 ***	0.22	0.144	0.296	<0.001 ***
troubled that child may not overcome FA	0.63	0.342	0.917	<0.001 ***	0.221	0.133	0.309	<0.001 ***
troubled with leaving child in care of others	0.593	0.294	0.892	<0.001 ***	0.162	0.07	0.254	<0.001 ***
troubled by other’s lack of knowledge of FA	0.33	0.046	0.614	0.023 *	0.093	0.005	0.181	0.038 *
troubled by sadness due to child burden	0.484	0.205	0.762	<0.001 ***	0.182	0.097	0.267	<0.001 ***
troubled by child’s attendance at school and extracurricular activities	0.64	0.359	0.922	<0.001 ***	0.186	0.099	0.273	<0.001 ***
troubled with concerns for child’s health	0.546	0.275	0.817	<0.001 ***	0.197	0.113	0.28	<0.001 ***
troubled with worry of inability to help in a reaction	0.464	0.197	0.731	<0.001 ***	0.165	0.083	0.248	<0.001 ***
troubled with worry child will not have normal life	0.493	0.241	0.744	<0.001 ***	0.164	0.087	0.242	<0.001 ***
troubled by concerns about child’s nutrition	0.358	0.091	0.625	0.009 **	0.104	0.022	0.186	0.013 *
troubled with child being near others while eating	0.689	0.414	0.963	<0.001 ***	0.216	0.132	0.301	<0.001 ***
troubled with fright about child having a reaction	0.526	0.249	0.803	<0.001 ***	0.165	0.079	0.25	<0.001 ***
composite	0.567	0.357	0.776	<0.001 ***	0.166	0.102	0.23	<0.001 ***

* *p* < 0.05; ** *p* < 0.01; *** *p* < 0.001.

## Data Availability

The raw data supporting the conclusions of this article will be made available by the authors on request.

## Abbreviations

The following abbreviations are used in this manuscript:

FA	Food Allergy
FAQL-PB	Food Allergy Quality of Life-Parental Burden
FAIM	Food Allergy Independent Measure
FAQLQ	Food allergy-related Quality of Life Questionnaire
Multi-FA	Multiple Food Allergy
NIAID	National Institute of Allergy and Infectious Disease

## Appendix A

**Table A1:** Demographic information for parent burden measure.

	Total (N = 729)		1 FA (N = 169)		2–3 FA (N = 314)		4–6 FA (N = 201)		7+ FA (N = 45)		*p* Value
	N	%	N	%	N	%	N	%	N	%	
Child Age											<0.001 ***
Mean Age (months) (s.d.)	70.4 (43.40)	-	57.8 (40.52)	-	71.3 (43.16)	-	75.7 (45.38)	-	87.8 (35.77)	-	
Sex											0.241
Male	451	61.9	97	57.4	190	60.5	133	66.2	31	68.9	
Female	278	38.1	72	42.6	124	39.5	68	33.8	14	31.1	
Race											0.026 *
White	407	55.8	91	53.8	178	56.7	119	59.2	19	42.2	
Black	258	35.4	58	34.3	102	32.5	73	36.3	25	55.6	
Asian	4	0.5	1	0.6	1	0.3	2	1	0	0	
Other	56	7.7	16	9.5	32	10.2	7	3.5	1	2.2	
Ethnicity											<0.001 ***
Not Hispanic	570	78.2	121	71.6	234	74.5	176	87.6	39	86.7	
Hispanic	128	17.6	39	23.1	67	21.3	18	9	4	8.9	
Household Income											0.183
Less than $100,000	329	45.1	81	47.9	140	44.6	81	40.3	27	60	
$100,000–$199,000	161	22.1	33	19.5	66	21	55	27.4	7	15.6	
More than $199,000	239	32.8	55	32.5	108	34.4	65	32.3	11	24.4	
Parental Education											0.432
Some High School	28	3.8	9	5.3	11	3.5	6	3	2	4.4	
High School Grad	57	7.8	11	6.5	25	8	14	7	7	15.6	
Some College or Higher	644	88.3	149	88.2	278	88.5	181	90	36	80	
Allergens											<0.001 ***
Egg	379	52	33	19.5	144	45.9	157	78.1	45	100	
Milk	222	30.5	14	8.3	71	22.6	100	49.8	37	82.2	
Peanut	477	65.4	50	29.6	214	68.2	169	84.1	44	97.8	
Treenut	446	61.2	42	24.9	180	57.3	180	89.6	44	97.8	
Finfish	124	17	4	2.4	32	10.2	57	28.4	31	68.9	
Shellfish	142	19.5	12	7.1	37	11.8	62	30.8	31	68.9	
Wheat	85	11.7	1	0.6	8	2.5	41	20.4	35	77.8	
Soy	96	13.2	0	0	16	5.1	44	21.9	36	80	
Sesame	140	19.2	6	3.6	30	9.6	71	35.3	33	73.3	
Other	141	19.3	7	4.1	36	11.5	71	35.3	27	60	
Comorbidities											<0.001 ***
Asthma	311	42.7	51	30.2	132	42	92	45.8	36	80	
Eczema	603	82.7	116	68.6	263	83.8	181	90	43	95.6	
Allergic Rhinitis	360	49.4	51	30.2	148	47.1	126	62.7	35	77.8	
Oral Allergy Syndrome	289	39.6	42	24.9	110	35	103	51.2	34	75.6	

The table above displays the demographic information for the parental burden questionnaire for the total 729 participants and then also broken down into categories of parents of children with 1 FA, 2–3 FA, 4–6 FA, and 7+ FA. * *p* < 0.05; ** *p* < 0.01; *** *p* < 0.001.

**Table A2:** Demographic Information for global health burden measure.

	Total (N = 346)		1 FA (N = 69)		2–3 FA (N = 143)		4–6 FA (N = 105)		7+ FA (N = 29)		*p* Value
	N	%	N	%	N	%	N	%	N	%	
Child Age											0.044 *
Mean Age (months) (s.d.)	69.2 (43.45)	-	62.9 (42.72)	-	66.9 (43.34)	-	71.1 (44.49)	-	88 (37.98)	-	
Sex											0.307
Male	216	62.4	37	53.6	89	62.2	71	67.6	19	65.5	
Female	130	37.6	32	46.4	54	37.8	34	32.4	10	34.5	
Race											0.09
White	219	63.3	42	60.9	88	61.5	75	71.4	14	48.3	
Black	109	31.5	22	31.9	43	30.1	29	27.6	15	51.7	
Asian	2	0.6	1	1.4	1	0.7	0	0	0	0	
Other	15	4.3	4	5.8	10	7	1	1	0	0	
Ethnicity											0.346
Not Hispanic	299	86.4	57	82.6	121	84.6	95	90.5	26	89.7	
Hispanic	36	10.4	8	11.6	19	13.3	7	6.7	2	6.9	
Household Income											0.127
Less than $100,000	130	37.6	30	43.5	55	38.5	29	27.6	16	55.2	
$100,000–$199,000	100	28.9	16	23.2	41	28.7	37	35.2	6	20.7	
More than $199,000	116	33.5	23	33.3	47	32.9	39	37.1	7	24.1	
Parental Education											0.215
Some High School	8	2.3	3	4.3	3	2.1	2	1.9	0	0	
High School Grad	23	6.6	3	4.3	10	7	5	4.8	5	17.2	
Some College or Higher	315	91	63	91.3	130	90.9	98	93.3	24	82.8	
Allergens											<0.001 ***
Egg	194	56.1	16	23.2	63	44.1	86	81.9	29	100	
Milk	110	31.8	4	5.8	27	18.9	54	51.4	25	86.2	
Peanut	238	68.8	20	29	103	72	87	82.9	28	96.6	
Treenut	225	65	18	26.1	82	57.3	96	91.4	29	100	
Finfish	65	18.8	2	2.9	19	13.3	24	22.9	20	69	
Shellfish	71	20.5	2	2.9	20	14	28	26.7	21	72.4	
Wheat	46	13.3	0	0	3	2.1	22	21	21	72.4	
Soy	56	16.2	0	0	7	4.9	24	22.9	25	86.2	
Sesame	83	24	3	4.3	13	9.1	45	42.9	22	75.9	
Other	71	20.5	4	5.8	16	11.2	33	31.4	18	62.1	
Comorbidities											<0.001 ***
Asthma	152	43.9	22	31.9	64	44.8	46	43.8	20	69	
Eczema	284	82.1	49	71	115	80.4	93	88.6	27	93.1	
Allergic Rhinitis	200	57.8	27	39.1	80	55.9	70	66.7	23	79.3	
Oral Allergy Syndrome	141	40.8	16	23.2	49	34.3	54	51.4	22	75.9	

The table above displays the demographic information for the PROMIS Pediatric Global Health for the total 346 participants and then also broken down into categories of children with 1 FA, 2–3 FA, 4–6 FA, and 7+ FA. * *p* < 0.05; ** *p* < 0.01; *** *p* < 0.001.

**Table A3:** Mean values of global health burden in children with single FA vs. multiple FA.

	Single FA Mean (SD)	Multi FA Mean (SD)	Bivariate *p* Value
PROMIS Global Health			
general health	1.76 (0.73)	1.90 (0.80)	0.123
quality of life	1.65 (0.67)	1.75 (0.76)	0.226
physical health	1.72 (0.77)	1.79 (0.78)	0.471
mental health	1.90 (0.80)	2.11 (0.89)	0.044 *
sadness	2.07 (0.66)	2.16 (0.67)	0.318
fun with friends	1.77 (0.76)	1.81 (0.76)	0.644
parents listen to ideas	1.78 (0.61)	1.83 (0.79)	0.54
composite	1.81 (0.51)	1.91 (0.56)	0.122

The table above displays the mean and *p*-values for single FA vs. multiple FA for the PROMIS Pediatric Global Health, which is scored from 1 to 5 with 333 observations. * *p* < 0.05; ** *p* < 0.01; *** *p* < 0.001.

**Table A4:** Global health burden for children with multiple FA regression analysis.

	Categorical B Coefficient	95% CI Lower	95% CI Upper	Categorical *p* value	Continuous B Coefficient	95% CI Lower	95% CI Upper	Continuous *p* Value
PROMIS Global Health								
general health	0.119	−0.154	0.391	0.393	0.037	−0.059	0.133	0.454
quality of life	0.23	−0.021	0.481	0.072	0.041	−0.048	0.13	0.363
physical health	0.171	−0.096	0.438	0.21	0.047	−0.048	0.141	0.33
mental health	−0.03	−0.332	0.272	0.845	−0.019	−0.126	0.088	0.722
sadness	−0.038	−0.264	0.189	0.745	−0.045	−0.124	0.035	0.273
fun with friends	0.101	−0.169	0.372	0.463	0.02	−0.076	0.115	0.684
parents listen to ideas	0.193	−0.066	0.452	0.145	0.101	0.009	0.192	0.032 *
composite	0.108	−0.085	0.301	0.272	0.03	−0.009	0.07	0.133

The table above displays the B coefficient and *p*-values for categorical (i.e., single FA vs. multiple FA) and continuous (i.e., specific number of FA) for the PROMIS Pediatric Global Health with 333 observations. * *p* < 0.05; ** *p* < 0.01; *** *p* < 0.001.
